# Activation of the Endoplasmic Reticulum Stress Response Impacts the NOD1 Signaling Pathway

**DOI:** 10.1128/IAI.00826-18

**Published:** 2019-07-23

**Authors:** Jonathan M. Mendez, Lakshmi Divya Kolora, James S. Lemon, Steven L. Dupree, A. Marijke Keestra-Gounder

**Affiliations:** a Department of Immunology and Microbiology, University of Colorado School of Medicine, Aurora, Colorado, USA; University of California, San Diego, School of Medicine

**Keywords:** ER stress, NF-κB, NOD1, *Salmonella*, colitis, inflammatory bowel disease

## Abstract

Nucleotide-binding oligomerization domain 1 (NOD1) is an intracellular pattern recognition receptor (PRR) responsible for sensing bacterial peptidoglycan fragments. Stimulation of NOD1 leads to a robust innate immune response via activation of the major transcription factor NF-κB. In addition to peptidoglycan sensing, NOD1 and the closely related PRR NOD2 have been linked to inflammation by responding to the endoplasmic reticulum (ER) stress-induced unfolded protein response (UPR). Here we show that differential ER stress induction renders cells more susceptible to Salmonella enterica serovar Typhimurium infection in a NOD1-dependent manner, measured by increased NF-κB activation and cytokine expression. In HeLa57A cells stably transfected with an NF-κB::luciferase reporter, we show that cells undergoing ER stress induced by thapsigargin display a significant increase in NF-κB activation in response to NOD1 stimulation by C12-iE-DAP (acylated derivative of the iE-DAP dipeptide [gamma-d-glutamyl-*meso*-diaminopimelic acid]) and the *S*. Typhimurium effector protein SopE. Tunicamycin-induced ER stress had no effect on NOD1-stimulated NF-κB activation. We further show that the mouse intestinal epithelial cell line MODE-K and RAW264.7 macrophages are more responsive to *Salmonella* infection when treated with thapsigargin but not with tunicamycin. These profound differences between thapsigargin- and tunicamycin-treated cells upon inflammation suggest that different components downstream of the UPR contribute to NOD1 activation. We found that the NOD1-induced inflammatory response is dependent on protein kinase R (PKR)-like endoplasmic reticulum kinase (PERK) activation in conjunction with stimulation of the inositol triphosphate receptor (IP3R). Together, these results suggest that differential UPR activation makes cells more responsive to bacterial infections in a NOD1-dependent manner.

## Abstract (precis)

Nucleotide-binding oligomerization domain 1 (NOD1) is an intracellular pattern recognition receptor (PRR) responsible for sensing bacterial peptidoglycan fragments. Stimulation of NOD1 leads to a robust innate immune response via activation of the major transcription factor NF-κB. In addition to peptidoglycan sensing, NOD1 and the closely related PRR NOD2 have been linked to inflammation by responding to the endoplasmic reticulum (ER) stress-induced unfolded protein response (UPR).

## INTRODUCTION

The innate immune system provides the first line of defense against invading pathogens like Salmonella enterica serovar Typhimurium by recognizing pathogen-associated molecular patterns (PAMPs). Detection of PAMPs by pattern recognition receptor (PRR) families such as the Toll-like receptors (TLRs) and the nucleotide-binding oligomerization domain (NOD)-like receptors (NLRs) activates signaling pathways that mediate inflammation, tissue repair, and microbial clearance (1). This is especially important in the gastrointestinal tract, where PRRs sense these PAMPs to protect against the invading pathogens while maintaining tolerance to commensal microbes (2). In addition to direct PAMP recognition, PRRs also play central roles in the detection of patterns of pathogenesis, defined as common pathogenic infection strategies to cause disease (3). NOD1 and NOD2, members of the NLR family, sense fragments of Gram-positive and Gram-negative bacterial peptidoglycans (4). In addition to their role as peptidoglycan sensors, NOD1 and NOD2 can detect perturbations in cellular processes such as the regulation of the actin cytoskeleton and disturbance in endoplasmic reticulum (ER) homeostasis (5–7). The ER is a highly dynamic organelle that exerts a major role in coordinating signaling pathways to ensure cellular homeostasis. The ER is the site of synthesis and folding of proteins; however, under different stressful pathological and physiological conditions, such as pathogen infections and perturbation of calcium homeostasis, the ER is unable to maintain homeostasis and activates the unfolded protein response (UPR) (8). Thapsigargin and tunicamycin are interchangeably used as common ER stress inducers. Tunicamycin inhibits protein glycosylation in the ER, leading to the accumulation of unfolded proteins and activation of the UPR. Thapsigargin inhibits the sarcoplasmic and endoplasmic reticulum Ca^2+^-ATPase (SERCA), leading to ER calcium depletion and increased cytosolic calcium concentrations (9). The influx of Ca^2+^ into the ER is mostly dependent on SERCA, and calcium release from the ER is mainly controlled by the 1,4,5-triphosphate (IP3) receptor (IP3R) (10). When calcium levels are lowered in the ER, the calcium-dependent chaperones lose their activity, resulting in an increase of unfolded proteins and activation of the UPR. Three transmembrane receptors, ATF6, protein kinase R (PKR)-like endoplasmic reticulum kinase (PERK), and IRE1α, are activated and regulate biological processes such as inhibition of protein translation, autophagy, and inflammation to reestablish cellular homeostasis. Under homeostatic conditions, the chaperone BiP, encoded by the *HSP5A* gene, is bound to these receptors, thereby preventing their activation. ER perturbation results in the release of BiP from ATF6, PERK, and IRE1α, resulting in dimerization and phosphorylation of these receptors to an active state. These receptors subsequently activate the transcription factors ATF6f, ATF4, and XBP1, respectively, which then bind to ER stress elements (ERSEs), which results in the transcription of UPR target genes such as *HSP5A*, *XBP1*, and *CHOP* (8). In addition, activated IRE1α induces XBP1-independent responses, which involves the recruitment of TRAF2 (tumor necrosis factor [TNF] receptor-associated factor 2) that in turn leads to the activation of mitogen-activated protein (MAP) kinases (MAPKs) and NF-κB and the subsequent production of proinflammatory cytokines such as interleukin-6 (IL-6) (8). NOD1 and NOD2 have been implicated in ER stress-induced inflammation, by linking the IRE1α/TRAF2 pathway of the UPR to NF-κB activation (7, 11). The link between UPR and NOD1/2 signaling is of particular interest in intestinal inflammation since mutations in genes associated with the UPR (*XBP1*) and innate immune signaling (*NOD2* and *IL23R*) have been associated with intestinal epithelial dysfunction and the onset and development of inflammatory bowel diseases (IBDs) such as Crohn’s disease (CD) (12). The exact cause of Crohn’s disease is currently unknown, but recent studies indicate that environmental factors and genetic defects contribute to the development of Crohn’s disease and that the interaction between these two factors is what triggers the pathology. Many enteric pathogens, including *S*. Typhimurium, have been implicated in the onset or development of IBD in susceptible individuals (13). Interestingly, the majority of the genetic defects associated with intestinal epithelial cell (IEC) function alone are not associated with the onset of IBD, highlighting the critical role and specificity of these genetic defects in combination with environmental triggers.

*S*. Typhimurium is a facultative, Gram-negative, intracellular bacterium that causes severe inflammation of the intestinal mucosal epithelium, resulting in gastroenteritis in humans (14). *S*. Typhimurium infections in mice are used as a model of intestinal inflammation. Pretreatment with streptomycin in mice subsequently infected with *S*. Typhimurium produces intestinal damage, including epithelial crypt loss, mucosal erosion, and neutrophil infiltration, similar to pathological changes observed in patients with inflammatory bowel disease (15). One of the main virulence factors required for gastroenteritis is type III secretion system 1 (T3SS-1) encoded on *Salmonella* pathogenicity island 1 (SPI1), which allows *S*. Typhimurium to invade epithelial cells and to induce a robust inflammatory response (14). Early innate immune responses during *S*. Typhimurium infection include the production of an array of chemokines and cytokines, such as IL-23 and IL-6 (16). Early IL-6 production was shown to be dependent on NOD1 and NOD2, and NOD2 stimulation resulted in the release of IL-23, indicating the significance of these PRRs in the production of IL-6 and IL-23 (17, 18).

The important connection between NOD1/2 signaling and IL-6 and IL-23 production during *S*. Typhimurium infection is evident. However, whether underlying ER stress and UPR activation influence the immune response during *S*. Typhimurium infection is currently unknown. Here we explore the role of differential UPR activation in *S*. Typhimurium-induced NOD1 activation. We demonstrate that PERK activation and stimulation of the IP3R increase the inflammatory response via a NOD1-dependent signaling pathway. Altogether, our results suggest that excessive inflammatory responses to bacterial infections are observed only in cells undergoing a specific ER stress response. This may explain why some but not all genetic defects causing ER stress and UPR activation have been linked to an increased susceptibility to bacterial infections and the onset/development of IBD. A better understanding of the influence of genetic variables affecting ER stress and the UPR and their interaction with environmental factors will help in the development of new therapeutics for the treatment of bacterial infections and IBD.

## RESULTS

### Distinct UPR activation increases *S*. Typhimurium-induced inflammatory responses.

**FIG 1:**
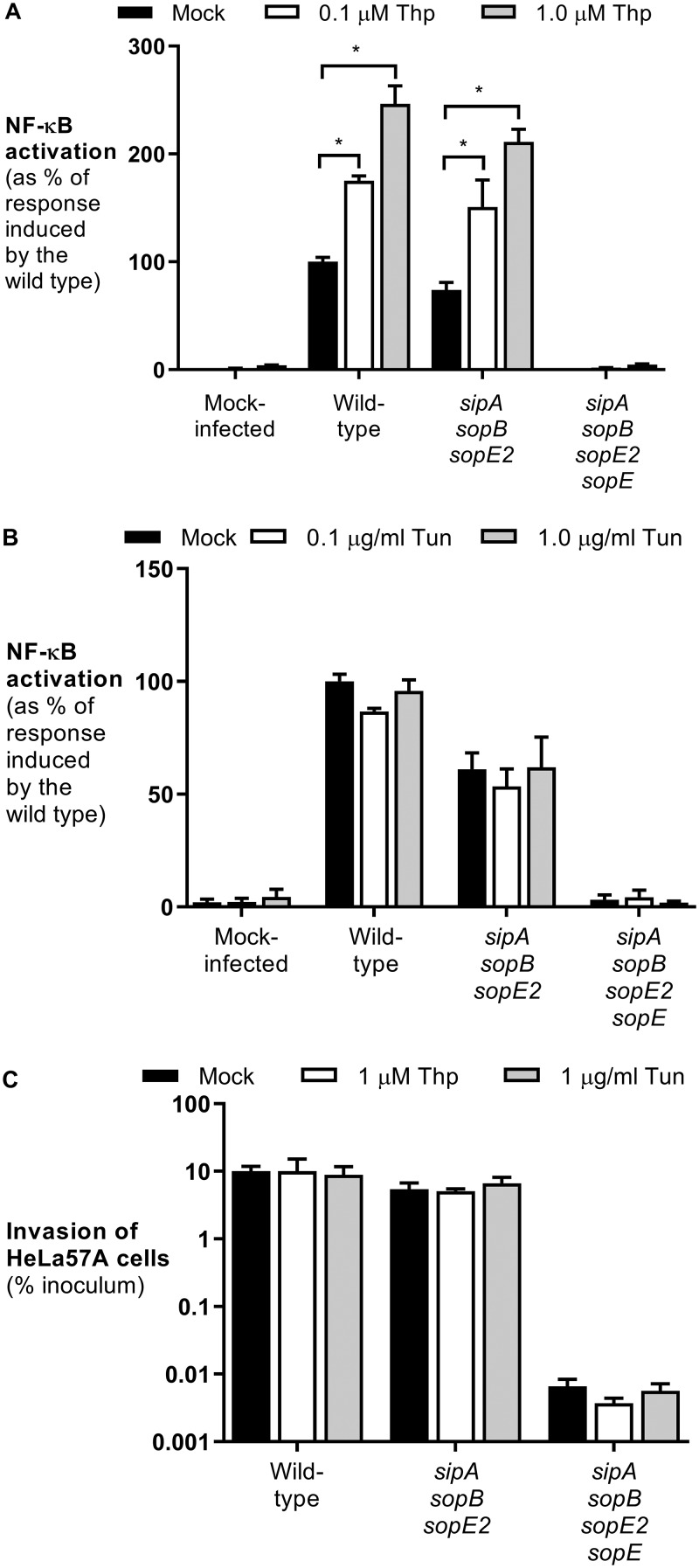
Thapsigargin increases NF-κB activation in response to *S*. Typhimurium independent of bacterial invasion. (A and B) HeLa57A cells were pretreated with thapsigargin (Thp) (0.1 and 1 μM) (A) or tunicamycin (Tun) (0.1 and 1 μg/ml) (B) and infected with the *S*. Typhimurium wild-type strain SL1344, the SopE-positive *sipA sopB sopE2* mutant strain, and the *sipA sopB sopE2 sopE* mutant strain. The response induced by *S*. Typhimurium wild-type strain SL1344 was set at 100%. Data represent the means ± standard errors from at least three independent experiments in triplicate. (C) Cells were infected at an MOI of 10 for 1 h, and extracellular bacteria were killed with gentamicin for 90 min. Recovered bacterial numbers were standardized to the number of bacteria in the inoculum. Data represent the means ± standard errors from three independent experiments in duplicate. One-way analysis of variance (ANOVA) followed by Dunnett’s multiple-comparison test was used to determine statistical significance. A *P* value of <0.05 was taken to be significant (indicated by an asterisk).

To determine whether ER stress renders cells more susceptible to *S*. Typhimurium infection, HeLa57A cells stably transfected with an NF-κB::luciferase reporter construct were treated with the ER stress inducers thapsigargin and tunicamycin prior to infection with *S*. Typhimurium. The NF-κB activity toward wild-type *S*. Typhimurium strain SL1344 was significantly greater in cells treated with increasing concentrations of thapsigargin (Fig. 1A). The proinflammatory response induced by *S*. Typhimurium is mostly dependent on the T3SS-1 effector proteins SipA, SopE, SopB, and SopE2 (6, 19). An *S*. Typhimurium mutant lacking these four effectors was unable to activate NF-κB, even in the presence of thapsigargin, suggesting that these effector proteins are sufficient for the increased NF-κB activation in cells undergoing ER stress (Fig. 1A). Previously, we showed that approximately 70% of the NF-κB activation induced by wild-type *S*. Typhimurium is induced by the effector protein SopE (6). Here we show that thapsigargin treatment of HeLa cells results in increased NF-κB activation induced by SopE (Fig. 1A). Interestingly, when cells were treated with the ER stress inducer tunicamycin, no significant effect was observed in *S*. Typhimurium-infected cells (Fig. 1B). The differences between the effects of thapsigargin and tunicamycin on *S*. Typhimurium-induced NF-κB activation were not the result of differences in cell invasion (Fig. 1C).

### Thapsigargin increases NOD1-dependent NF-κB activation.

**FIG 2:**
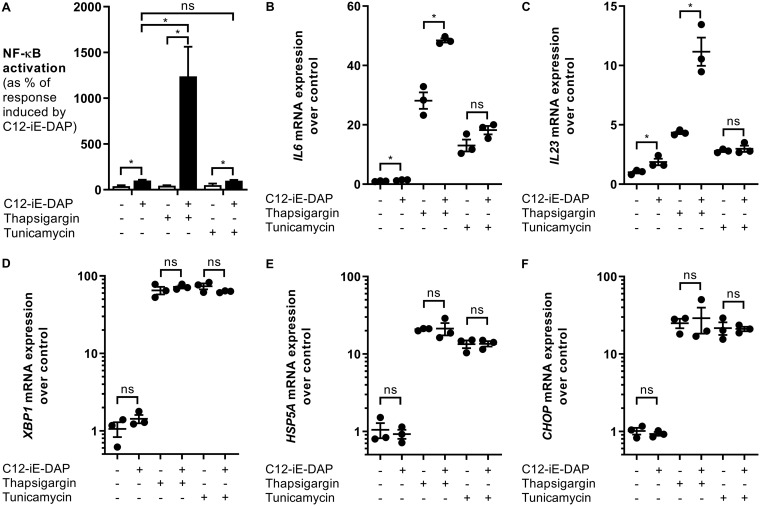
Thapsigargin increases the NOD1-dependent inflammatory response. HeLa57A cells were stimulated with the NOD1 ligand C12-iE-DAP (10 μg/ml) in combination with thapsigargin (1 μM) or tunicamycin (1 μg/ml). (A) Luciferase activity was quantified 5 h after stimulation to determine NF-κB levels. The response induced by C12-iE-DAP alone was set at 100%. Data represent means ± standard errors from at least three independent experiments in triplicate. (B to F) RNA was extracted, and RT-PCR was performed to determine the expression levels of *Il6* (B), *Il23* (C), *Xbp1* (D), *Hsp5a* (E), and *Chop* (F) mRNAs. Shown are data from one representative experiment in triplicate of three independent experiments. One-way ANOVA followed by a Bonferroni multiple-comparison test was used to determine statistical significance in panel A. Two-tailed paired Student’s *t* test was used to confirm statistical significance in panels B to F. A *P* value of <0.05 was taken to be significant. ns, not significant.

**FIG 3:**
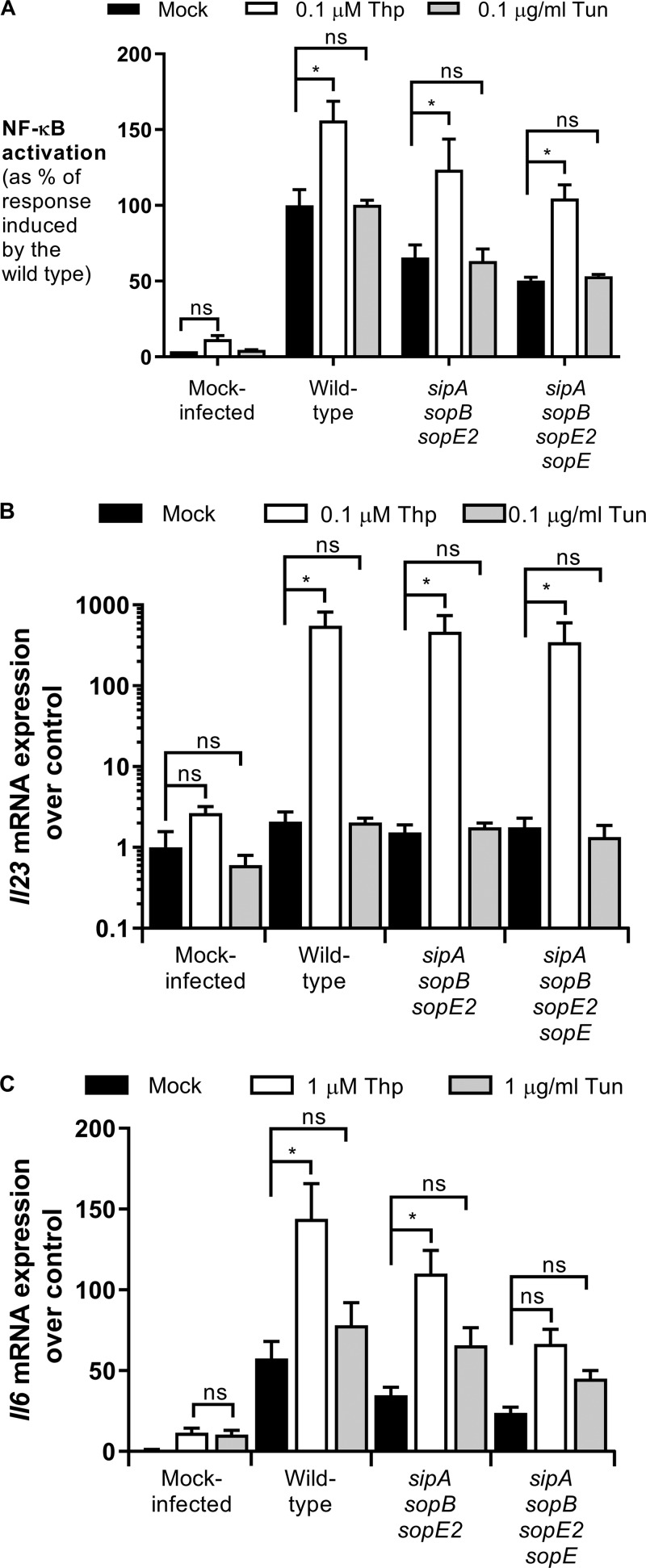
Thapsigargin increases *S*. Typhimurium-induced inflammatory responses in model intestinal epithelial cells and macrophages. (A) RAW264.7 macrophages stably expressing an NF-κB–luciferase reporter were pretreated with thapsigargin (0.1 μM) or tunicamycin (0.1 μg/ml) and infected with the *S*. Typhimurium wild-type strain SL1344, the SopE-positive *sipA sopB sopE2* mutant strain, and the *sipA sopB sopE2 sopE* mutant strain. The response induced by *S*. Typhimurium wild-type strain SL1344 was set at 100%. Data represent the means ± standard errors from at least three independent experiments in triplicate. (B) RNA was extracted, and RT-PCR was performed to determine the expression level of *Il23*. Data represent means ± standard errors from three independent experiments in duplicate. One-way ANOVA followed by Dunnett’s multiple-comparison test was used to determine statistical significance. A *P* value of <0.05 was taken to be significant. (C) MODE-K cells were pretreated with thapsigargin (1 μM) or tunicamycin (1 μg/ml) and infected with the *S*. Typhimurium wild-type strain SL1344, the SopE-positive *sipA sopB sopE2* mutant strain, and the *sipA sopB sopE2 sopE* mutant strain. RNA was extracted, and RT-PCR was performed to determine the expression level of *Il6*. Data represent means ± standard errors from at least three independent experiments in duplicate. One-way ANOVA followed by Dunnett’s multiple-comparison test was used to determine statistical significance. A *P* value of <0.05 was taken to be significant.

SopE has been shown to activate the PRR NOD1; therefore, we hypothesized that the increased NF-κB activation was mediated by NOD1 stimulation (6). To test this, we treated cells with thapsigargin or tunicamycin and subsequently stimulated HeLa57A cells with the NOD1 ligand C12-iE-DAP (acylated derivative of the iE-DAP dipeptide [gamma-d-glutamyl-*meso*-diaminopimelic acid]). Only in the cells pretreated with thapsigargin, and not in those treated with tunicamycin, did we observe a significant increase in NF-κB activation in response to NOD1 stimulation, indicating the importance of NOD1 activation in cells undergoing differential ER stress (Fig. 2A). NF-κB is a major transcription factor important for the upregulation of a large array of genes, including the cytokines IL-6 and IL-23. NOD1 activation with C12-iE-DAP resulted in increased transcription of *Il6* and *Il23* in thapsigargin-treated cells compared with tunicamycin-treated cells (Fig. 2B and C). These profound differences between thapsigargin and tunicamycin were not caused by a lack of UPR induction since both compounds significantly increased the transcription of the UPR target genes *Xbp1*, *Hsp5a*, and *Chop* in HeLa57A cells (Fig. 2D to F). To test whether thapsigargin pretreatment also augmented the inflammatory response in macrophages, we infected RAW264.7 murine macrophages that are stably transfected with an NF-κB luciferase reporter with our *S*. Typhimurium strains (Fig. 3A). Similarly, NF-κB activation was significantly increased when the cells were pretreated with thapsigargin and not with tunicamycin. In contrast to HeLa57A cells, the *sipA sopB sopE2 sopE* mutant was able to activate NF-κB in RAW264.7 cells, which is likely mediated by TLR activation. The mutant strains induced lower NF-κB activation than the wild-type strain, indicating that the effector proteins contribute to the activation of the innate immune response in macrophages. NF-κB activation correlated with a robust increase in *Il23* expression in macrophages, the major source of IL-23 production (Fig. 3B) (20). Although tunicamycin-treated macrophages had reduced (∼3-fold) expression of the UPR target genes compared to thapsigargin-treated macrophages (see Fig. S2 in the supplemental material), only an ∼2-fold increase in *Il23* expression was observed in these cells, compared to an ∼550-fold increase with thapsigargin treatment. Intestinal epithelial cells (IECs) play an essential role in the innate immune response against invading *Salmonella*. Therefore, we treated the mouse small IEC line MODE-K with the ER stress inducers and subsequently infected the cells with the *S*. Typhimurium strains (21). Consistently, thapsigargin-pretreated MODE-K cells showed significantly increased *Il6* expression when infected with *S*. Typhimurium. Similar to the macrophages, infection with the *sipA sopB sopE2 sopE* mutant led to *Il6* expression, most likely induced by TLR activation (Fig. 3C). Although thapsigargin induced increased *Il6* levels in *sipA sopB sopE2 sopE* mutant-infected cells, this was not statistically significant. In intestinal epithelial cells, thapsigargin and tunicamycin induced similar expression levels of the UPR target genes *Xbp1*, *Hsp5a*, and *Chop* (Fig. S3). Together, these results suggest that ER stress induced by thapsigargin, but not by tunicamycin, renders cells more responsive to *Salmonella* infection.

### Activation of the IP3R is required for the increased NOD1 response.

**FIG 4:**
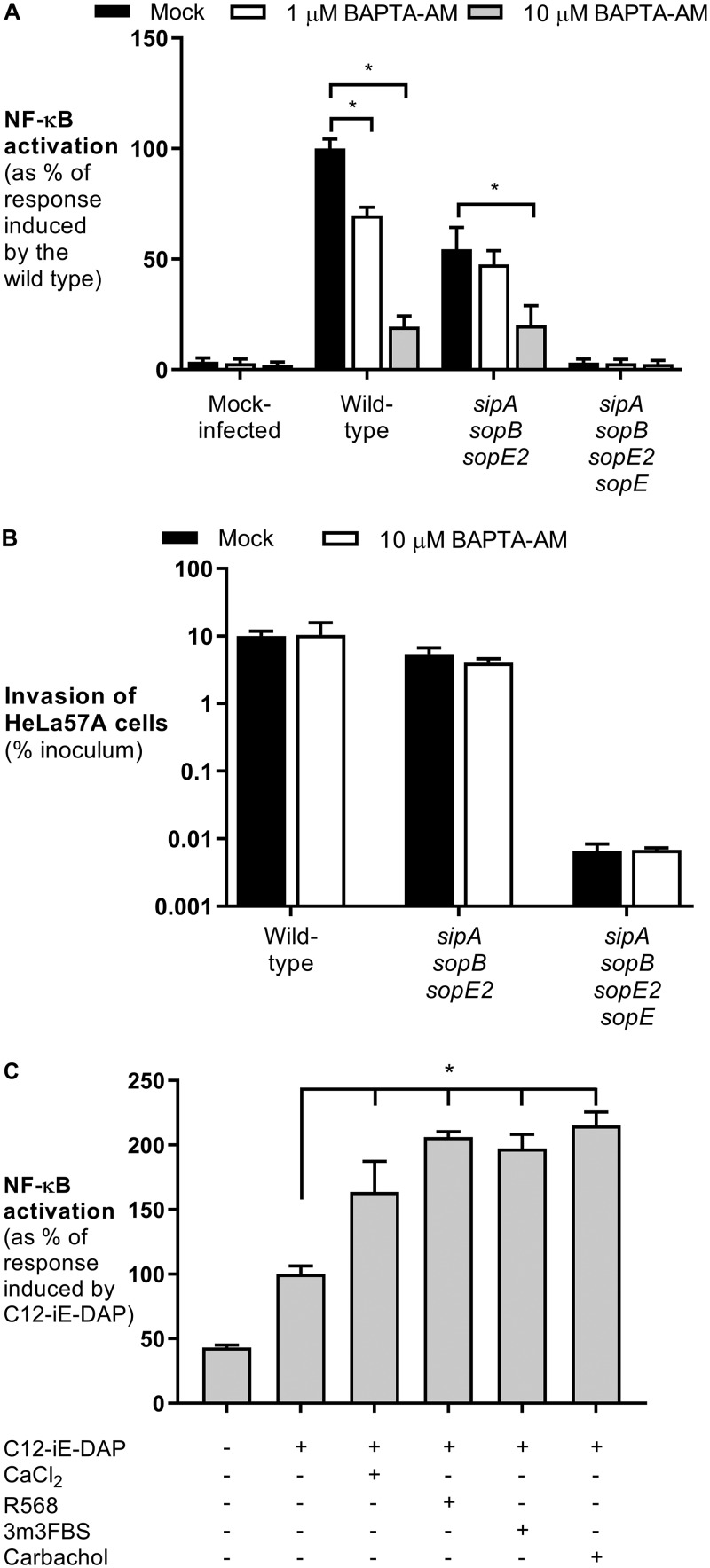
Activation of the IP3R and calcium increase the NOD1 response. (A) HeLa57A cells were pretreated with BAPTA-AM (10 μM) and infected with the *S*. Typhimurium wild-type strain SL1344, the SopE-positive *sipA sopB sopE2* mutant strain, and the *sipA sopB sopE2 sopE* mutant strain. The response induced by *S*. Typhimurium wild-type strain SL1344 was set at 100%. Data represent the means ± standard errors from at least three independent experiments in triplicate. (B) Cells were infected at an MOI of 10 for 1 h, and extracellular bacteria were killed with gentamicin for 90 min. Recovered bacterial numbers were standardized to the number of the bacteria in the inoculum. Data represent the means ± standard errors from three independent experiments in duplicate. (C) HeLa57A cells were stimulated with the NOD1 ligand C12-iE-DAP (10 μg/ml) in combination with extracellular CaCl_2_ (1 mM), the CaSR agonist R568 (10 μM), the PLC activator 3m3FBS (50 μM), and the IP3R agonist carbachol (5 mM). Luciferase activity was quantified 5 h after stimulation to determine NF-κB levels. The response induced by C12-iE-DAP alone was set at 100%. Data represent means ± standard errors from at least three independent experiments in triplicate. One-way ANOVA followed by Dunnett’s multiple-comparison test was used to determine statistical significance. A *P* value of <0.05 was taken to be significant.

Inhibition of SERCA by thapsigargin leads to ER calcium depletion, suggesting that alterations in calcium concentrations are required for NOD1 signaling. HeLa cells and intestinal epithelial cells infected with *S*. Typhimurium displayed a significant increase in the intracellular Ca^2+^ concentration (22, 23). Here we show that chelation of Ca^2+^ with BAPTA-AM [1,2-bis(2-aminophenoxy)ethane-*N,N,N′,N′*-tetraacetic acid tetrakis(acetoxymethyl ester)] reduced NF-κB activation induced by wild-type *S*. Typhimurium and the *sipA sopB sopE2* mutant (Fig. 4A). Similar to thapsigargin and tunicamycin, the addition of BAPTA-AM had no effect on bacterial invasion (Fig. 4B). To investigate the importance of calcium signaling in NOD1 activation, we added 1 mM CaCl_2_ to the growth medium of HeLa57A cells and subsequently stimulated the cells with C12-iE-DAP. The addition of extracellular calcium significantly increased NOD1-dependent NF-κB activation (Fig. 4C). Extracellular calcium is sensed by the calcium-sensing receptor (CaSR), resulting in a conformational change (24). This change activates phospholipase C (PLC) that catalyzes the cleavage of phosphatidylinositol 4,5-biphosphate (PIP2) into diacylglycerol (DAG) and inositol 1,4,5-triphosphate (IP3). IP3, in turn, is required to activate the ER membrane IP3 receptor (IP3R), which releases calcium from the ER into the cytosol (24). The activation of CaSR with the agonist R568 significantly increased C12-iE-DAP-induced NF-κB activity, suggesting a role for the IP3 receptor (Fig. 4C). To confirm these findings, we treated HeLa57A cells with the PLC agonist 3m3FBS {2,4,6-trimethyl-*N*-[3-(trifluoromethyl)phenyl]benzenesulfonamide} and the IP3R agonist carbachol prior to NOD1 stimulation (Fig. 4C). Similarly, NF-κB activity was significantly increased in the presence of these agonists, indicating the importance of calcium flux from the ER via IP3R in activating the NOD1 signaling pathway.

### IP3R stimulation and the UPR synergize to elicit NOD1-dependent responses.

**FIG 5:**
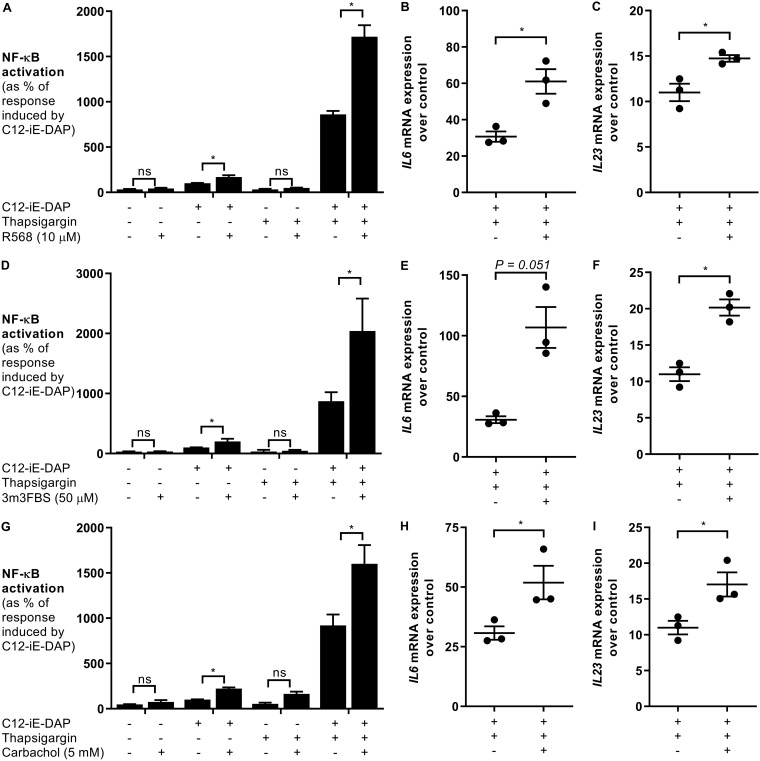
IP3R stimulation and ER stress increase NOD1-dependent responses. HeLa57A cells were stimulated with the NOD1 ligand C12-iE-DAP (10 μg/ml) in combination with thapsigargin (1 μM) and the CaSR agonist R568 (10 μM) (A to C), the PLC activator 3m3FBS (50 μM) (D to F), and the IP3R agonist carbachol (5 mM) (G to I). (A, D, and G) Luciferase activity was quantified 5 h after stimulation to determine NF-κB levels. The response induced by C12-iE-DAP alone was set at 100%. Data represent means ± standard errors from at least three independent experiments in triplicate. (B, C, E, F, H, and I) RNA was extracted, and RT-PCR was performed to determine the expression of *Il6* (B, E, and H) and *Il23* (C, F, and I) over the levels in unstimulated control samples. Data represent means ± standard errors from three independent experiments in duplicate. Two-tailed paired Student’s *t* test was used to confirm statistical significance. A *P* value of <0.05 was taken to be significant.

Activation of CaSR, PLC, and IP3R is a UPR-independent signaling event that resulted in a significant increase (∼200%) in the NOD1 response (Fig. 3C). Thapsigargin treatment, on the other hand, increased the NOD1 response to approximately 1,000% of the C12-iE-DAP response alone (Fig. 2A). Thapsigargin activates IP3R-regulated calcium flux in addition to the activation of the UPR, suggesting that both calcium flux and UPR activation are required for optimal NOD1 activation (25). To test this idea, we treated HeLa57A cells with thapsigargin and the CaSR activator R568 (Fig. 5A to C), the PLC agonist 3m3FBS (Fig. 5D to F), and the IP3 agonist carbachol (Fig. 5G to I). Together with C12-iE-DAP, activation of the CaSR/PLC/IP3R pathway in conjunction with UPR activation resulted in an even further increase in NF-κB activation, indicating that UPR activation and IP3R stimulation synergize to elicit stronger NOD1-induced inflammatory responses (Fig. 5). Corresponding with the increase in NF-κB activation, costimulation with agonists of the CaSR/PLC/IP3R pathway, C12-iE-DAP and thapsigargin, significantly increased *Il6* and *Il23* expression (Fig. 5).

### PERK activation is responsible for the increased NOD1 response.

**FIG 6:**
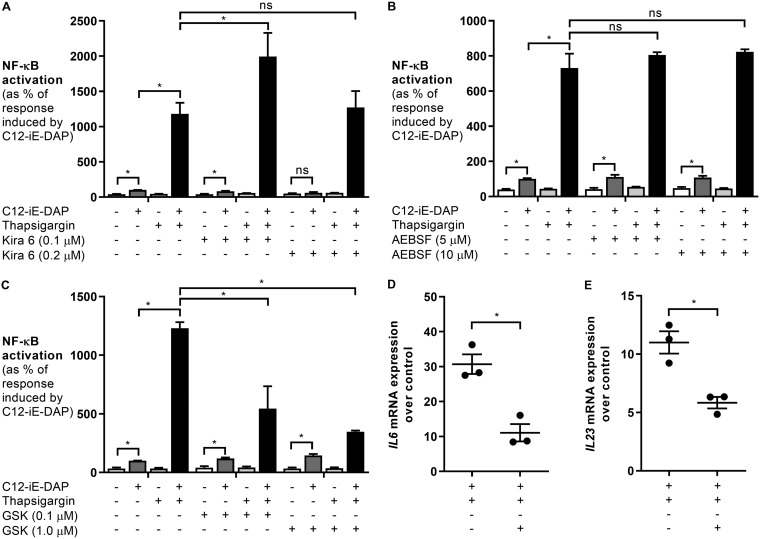
Inhibition of PERK decreases C12-iE-DAP- and ER stress-induced responses. HeLa57A cells were stimulated with the NOD1 ligand C12-iE-DAP (10 μg/ml) in combination with thapsigargin (1 μM) and the IRE1α inhibitor KIRA6 (0.1 and 0.2 μM) (A), the ATF6 inhibitor AEBSF (5 and 10 μM) (B), and the PERK inhibitor GSK2656157 (0.1 and 1 μM) (C to E). (A to C) Luciferase activity was quantified 5 h after stimulation to determine NF-κB levels. The response induced by C12-iE-DAP alone was set at 100%. Data represent means ± standard errors from at least three independent experiments in triplicate. One-way ANOVA followed by a Bonferroni multiple-comparison test was used to determine statistical significance. A *P* value of <0.05 was taken to be significant. (D and E) RNA was extracted, and RT-PCR was performed to determine the expression of *Il6* (D) and *Il23* (E) over the levels in unstimulated control samples. Data represent means ± standard errors from three independent experiments in duplicate. Two-tailed paired Student’s *t* test was used to confirm statistical significance. A *P* value of <0.05 was taken to be significant.

UPR activation involves the three transmembrane receptors IRE1α, ATF6, and PERK (8). To elucidate if any of these receptors synergize with IP3R to activate NOD1-dependent NF-κB responses, we incubated HeLa57A cells with specific inhibitors of IRE1α, ATF6, and PERK prior to stimulation with thapsigargin and C12-iE-DAP. Treatment of the cells with KIRA6, an inhibitor of IRE1α, had no effect on NF-κB activation (Fig. 6A) (26). The highest concentration of KIRA6 used in this study (0.2 μM) inhibited the C12-iE-DAP response, indicating that at this concentration, KIRA6 may have off-target effects, since the C12-iE-DAP-induced NOD1 response is independent of ER stress (7). Even with the higher concentration of KIRA6, there was no inhibition detected in NF-κB activation in cells treated with thapsigargin and C12-iE-DAP, suggesting that IRE1α is not responsible for the synergistic effect. Similarly, treatment with 4-(2-aminoethyl)benzenesulfonyl fluoride hydrochloride (AEBSF), an inhibitor of ATF6, had no inhibitory effect on thapsigargin- and C12-iE-DAP-induced NF-κB activation (Fig. 6B) (27). Treatment with the PERK inhibitor GSK2656157, however, showed dose-dependent inhibition, indicating that PERK activation increases NOD1-dependent responses (Fig. 6C to E) (28). The C12-iE-DAP response was not affected by the inhibitor, suggesting that there were no off-target effects. Altogether, our results suggest that PERK activation and calcium flux from the IP3R play an important role in mediating the NOD1 immune response (Fig. S1).

## DISCUSSION

Our results show that underlying ER stress and activation of the UPR increase the inflammatory response during *S*. Typhimurium infection dependent on SopE-induced NOD1 signaling, suggesting that inhibition of ER stress will reduce intestinal inflammation. Indeed, oral treatment of mice with the bile salt tauroursodeoxycholic acid (TUDCA), an ER stress inhibitor, ameliorated inflammation in mouse models of IBD (29, 30). Treatment of mice with TUDCA during *Salmonella* infection resulted in increased bacterial numbers in the intestine, liver, and spleen (31), suggesting that TUDCA treatment reduced the ER stress-induced NOD1/2 inflammatory response to *S*. Typhimurium, which impeded control of bacterial invasion and dissemination. In fact, NOD1/2 was previously shown to restrict *S*. Typhimurium infection in mice (32). *Nod1* and *Nod2* double-deficient mice infected with *S*. Typhimurium have reduced inflammatory responses but increased bacterial numbers in intestinal tissues (32). *S*. Typhimurium-induced ER stress activates the alternative secretory autophagy pathway in Paneth cells to secrete the antimicrobial lysozyme into the intestinal lumen to mediate bacterial killing of *Salmonella* (31), which is likely due to NOD signaling, as the NOD1/2 adapter RIP2 is indispensable for lysozyme sorting in Paneth cells (33). These and our results suggest that alterations in the UPR render intestinal cells more susceptible to bacterial infections in a NOD1/2-dependent manner, causing aberrant intestinal inflammation.

We show that treatments of cells with different ER stress inducers have differential effects on the response against *S*. Typhimurium, indicating that not all ER stress inducers activate the same downstream signaling pathways. Interestingly, not all genetic defects associated with an increased risk of development of CD display colitis symptoms in mouse models that are used to study intestinal inflammatory disorders. *Nod2^−/−^* mice are not more susceptible to dextran sodium sulfate (DSS)-induced colitis than wild-type controls (34); however, *Nod2^−/−^* mice develop signs of colitis in the T cell-induced mucosal damage model (35). These profound differences in disease outcomes in mice with the same genetic background underscore that distinct environmental triggers and the specific signaling pathways that they activate have a key contribution to the onset of disease in susceptible individuals.

Our results indicate that the PERK branch of the UPR is required for the increased NOD1-dependent inflammatory response. Similarly, PERK activation was also required for the ER stress-induced secretory autophagy pathway in *S*. Typhimurium-infected mice (31). Both tunicamycin and thapsigargin can activate the PERK pathway, suggesting that differential PERK activation leads to the transcription of different genes responsible for the increase in NOD1 signaling. Tunicamycin-induced *CHOP* expression, which is downstream of PERK activation, was shown to be dependent on p38 MAPK, whereas p38 MAPK inhibition in thapsigargin-treated cells had no effect on *CHOP* induction (36). Additionally, PERK-dependent induction of c-*myc* expression is specific to thapsigargin but not tunicamycin (37). Which protein(s) dependent on thapsigargin-induced PERK activation is responsible for the increased NOD1 response remains to be elucidated and will be the focus of future studies. Identifying the signaling components that orchestrate ER stress-induced responses resulting in increased susceptibility to bacterial infections may help in the development of new therapeutics specifically designed to target these pathways.

Activation of TLRs, specifically TLR2 and TLR4, has been reported to activate the IRE1α branch of the UPR but not the ATF6 or PERK pathway (38). The transcription factor XBP1, which is downstream of IRE1α, was also activated upon stimulation of TLR2 and TLR4. Consistent with our findings, activation of ER stress acted in synergy with TLR activation for cytokine production (38). Interestingly, our findings suggest that the synergy between ER stress and NOD1 activation is dependent on PERK activation. Infection of myeloid cells with Chlamydia trachomatis resulted in the expression of the transcription factor CHOP, which is downstream of PERK and was shown to bind to the promoter region of *Il23*, thereby augmenting its transcription (39). *Il23* expression is also dependent on NF-κB activation, suggesting that NF-κB and CHOP collaborate to enhance *Il23* expression. In MODE-K intestinal epithelial cells and RAW264.7 macrophages, we observed *Il6* and *Il23* expression, respectively, when cells were infected with the *sipA sopB sopE2 sopE* mutant strain. This increase in cytokine expression most likely resulted from TLR activation. Since Hela57A cells are not responsive to TLR and NOD2 activation, the synergistic effect of thapsigargin on the inflammatory response in HeLa57A cells is NOD1 mediated. Interestingly, in all cell lines, the synergistic response was observed only in cells treated with thapsigargin and not in those treated with tunicamycin, despite similar expression levels of the UPR target genes. These results suggest that differential ER stress activation (thapsigargin versus tunicamycin) impacts the response to *Salmonella* infection and NOD1 activation.

Our data show that the activation of the CaSR/PLC/IP3R signaling pathway increases NOD1 inflammatory responses. Interestingly, inhibition of CaSR with the antagonist NPS2143 resulted in reduced phosphorylation of p65, a subunit of NF-κB, in muramyl dipeptide (MDP)-stimulated cells, supporting our finding that CaSR stimulation increases NF-κB activity in response to peptidoglycan fragments (40). The decrease in the NOD2 response is possibly caused by reduced peptidoglycan uptake via micropinocytosis dependent on Gα, PLCγ, and the small Rho GTPases Rac1 and Cdc42, although direct evidence of impaired MDP uptake in NPS2143-treated cells was missing (40). CaSR activation promotes the activation of Rac1 and/or Cdc42, leading to the induction of membrane ruffles. We have previously shown that SopE-induced Rac1 activation and subsequent membrane ruffling recruit NOD1 to the cell membrane to induce an inflammatory response (7). A possible mechanism of how CaSR activation increases NOD activation is via the activation of Rac1 leading to the formation of membrane ruffles and NOD1/2 recruitment to the cell membrane (5, 6, 41, 42). In addition, it was shown that Rac1 can activate PLCβ to hydrolyze PIP2 into DAG and IP3 (43). This suggests that the activation of Rac1 by SopE or CaSR may lead to the activation of PLC and the production of the second messenger IP3 to stimulate the IP3R, thereby increasing the NOD1 response. Additionally, a study from 2008 showed that stimulation of macrophages and dendritic cells with peptidoglycan resulted in the phosphorylation of PLCγ2 (44). PLCγ2 knockdown in RAW264.7 cells stimulated with peptidoglycan led to reduced IL-6 and TNF-α production, supporting our findings about the importance of PLC activity for NOD signaling (44, 45). The exact underlying mechanism of how CaSR/PLC/IP3R activation, Rac1/Cdc42-mediated membrane ruffling, disruption of calcium homeostasis, and increased NOD1/2 signaling are linked remains to be elucidated.

Increased NOD signaling with underlying ER stress might be a common mechanism in other chronic inflammatory diseases associated with ER stress and UPR activation, including ankylosing spondylitis (AS) and type 2 diabetes (T2D) (46, 47). Peripheral blood mononuclear cells (PBMCs) of patients with active AS were hyperresponsive to MDP stimulation, and increased *Il23*, *Il17*, and *Il1b* expression was demonstrated (48). Monocytes isolated from T2D patients had significantly increased production of IL-6 and TNF-α in response to NOD1 and NOD2 stimulation compared to monocytes from healthy control patients (49). Interestingly, mutations in the IL-23/IL-17 axis have been linked to an increased susceptibility to AS, and IL-17 levels are increased in patients with T2D (50–52). Although ER stress has been implicated in AS and diabetes, it remains to be elucidated whether the increased inflammatory response after NOD1 and NOD2 stimulation is mediated by UPR activation. Here we present data that cells become hyperresponsive to NOD1 stimulation dependent on changes in calcium signaling and PERK activation. The connection between ER stress and the NOD/IL-23 axis may be of importance not just during *S*. Typhimurium infection and IBD but also in other chronic inflammatory diseases, including AS and T2D.

## MATERIALS AND METHODS

### Cells and reagents.

The HeLa57A cell line, stably transfected with an NF-κB luciferase reporter construct (53), was routinely propagated in Dulbecco’s modified Eagle’s medium (DMEM) (Gibco) with 5% fetal bovine serum (FBS) (Gibco) at 37°C in a 5% CO_2_ atmosphere. The murine small intestinal cell line MODE-K was cultured in DMEM with 2% sodium pyruvate, 1% nonessential amino acids, and 5% FBS (Gibco). RAW264.7 macrophages were propagated in DMEM with 10% FBS. Salmonella enterica serovar Typhimurium strain SL1344 was used as the wild-type isolate. *S*. Typhimurium SL1344 Δ*sopE* Δ*sipA sopB*::MudJ *sopE2*::pSB1039 and SL1344 Δ*sipA sopB*::MudJ *sopE2*::pSB1039 have been described previously (6). C12-iE-DAP was purchased from InvivoGen. Thapsigargin, tunicamycin, carbachol, 3m3FBS, KIRA6, and BAPTA-AM were purchased from Sigma-Aldrich. R568 was purchased from Tocris Bioscience, AEBSF was purchased from Fisher, and GSK2656157 was purchased from Calbiochem.

### Luciferase assays.

HeLa57A and RAW264.7 cells were seeded at 5 × 10^4^ cells per well in a 48-well tissue culture plate in DMEM plus FBS. Cultures of *S*. Typhimurium strains grown overnight were diluted 1 in 100 and grown for 3 h at 37°C. Prior to infection with the *S*. Typhimurium strains (multiplicity of infection [MOI] of 10 for HeLa57A and MOI of 1 for RAW264.7 cells), HeLa57A and RAW264.7 cells were pretreated with thapsigargin, tunicamycin, or BAPTA-AM. The plate was incubated for 1 h at 37°C, after which the medium was replaced with fresh cell culture medium, and the plate was incubated at 37°C for an additional 4 h. Cells stimulated with the indicated combinations of chemical compounds prior to C12-iE-DAP (10 μg/ml) stimulation were incubated for 5 h at 37°C. Next, the cells were lysed with 1× reporter lysis buffer (Promega), and firefly luciferase was measured with the luciferase assay system (Promega).

### Invasion assay.

HeLa57A cells were seeded in a 24-well tissue culture plate at 10^5^ cells per well. Cultures of *S*. Typhimurium strains grown overnight were diluted 1 in 100 and grown for 3 h at 37°C. HeLa57A cells were infected at an MOI of 10, and the plate was incubated for 1 h at 37°C to allow invasion. The cells were washed three times with Dulbecco’s phosphate-buffered saline (DPBS) (Gibco) to remove extracellular bacteria. Next, cells were incubated at 37°C for 90 min in DMEM with 5% FBS and gentamicin (0.1 mg/ml; Gibco). The cells were washed in DPBS and lysed with 0.5 ml of 1% Triton X-100, and the lysates were transferred to Eppendorf tubes. The wells were washed with 0.5 ml phosphate-buffered saline (PBS) and transferred to Eppendorf tubes. Serial dilutions were plated on LB plates with the appropriate antibiotics to count the intracellular bacteria. Invasiveness was calculated as the percentage of recovered bacteria compared to the inoculum.

### Real-time PCR.

Hela57A cells were seeded in 6-well tissue culture plates at 10^6^ cells per well. Cells were stimulated for 5 h with C12-iE-DAP alone or in combination with thapsigargin or tunicamycin. RAW264.7 and MODE-K cells were seeded in a 24-well tissue culture plate at 10^5^ cells/well. Cells were pretreated with thapsigargin or tunicamycin and infected with the *S*. Typhimurium strains (MOI of 1 for RAW264.7 cells and MOI of 10 for MODE-K cells). The plate was incubated for 1 h at 37°C, after which the medium was replaced with fresh cell culture medium, and the plate was incubated at 37°C for an additional 4 h. RNA was extracted using Tri reagent (Molecular Research Center) according to the instructions of the manufacturer. Reverse transcription (RT) was performed on 1 μg of DNase-treated RNA with TaqMan reverse transcription reagent (Applied Biosystems). Real-time RT-PCR was performed using SYBR green (Applied Biosystems) and the Quantstudio 7 Flex real-time PCR system (Applied Biosystems). The fold change in mRNA levels was determined using the comparative threshold cycle (ΔΔ*C_T_*) method. Target gene transcription was normalized to the levels of glyceraldehyde-3-phosphate dehydrogenase (*GAPDH*) mRNA.

## Supplementary Material

Supplemental file 1

Supplemental file 2

Supplemental file 3
