# Supplementary material for: Activation of the Endoplasmic Reticulum Stress Response Impacts the NOD1 Signaling Pathway
Source: Infect Immun. 2019 Jul 23;87(8):e00826-18. doi: 10.1128/IAI.00826-18 (PMC6652781; doi:10.1128/IAI.00826-18)
Supplement: Supplemental file 1 [file IAI.00826-18-s0001.pdf]

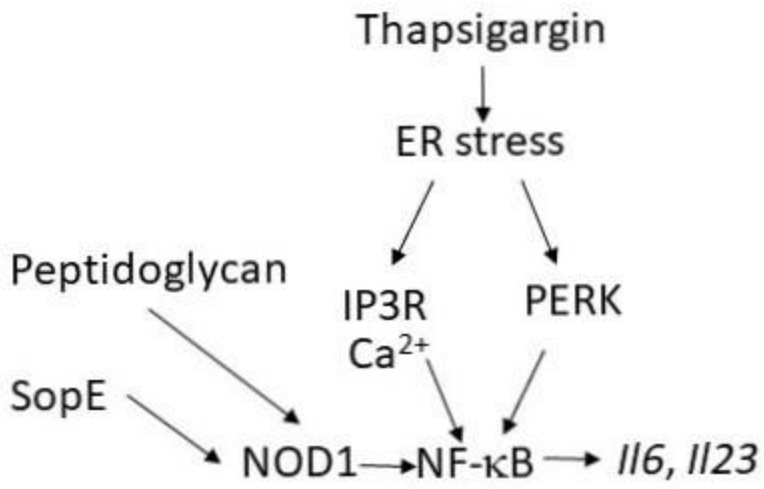

Figure S1. Thapsigargin-induced ER stress synergizes with NOD1 activation. Activation of NOD1 by SopE or peptidoglycan results in NF-κB activation and expression of *Il6* and *Il23*. The NOD1-mediated inflammatory response is synergistically increased via activation of PERK and IP3R induced by the ER stress inducer thapsigargin.
