# Supplementary material for: Activation of the Endoplasmic Reticulum Stress Response Impacts the NOD1 Signaling Pathway
Source: Infect Immun. 2019 Jul 23;87(8):e00826-18. doi: 10.1128/IAI.00826-18 (PMC6652781; doi:10.1128/IAI.00826-18)
Supplement: Supplemental file 3 [file IAI.00826-18-s0003.pdf]

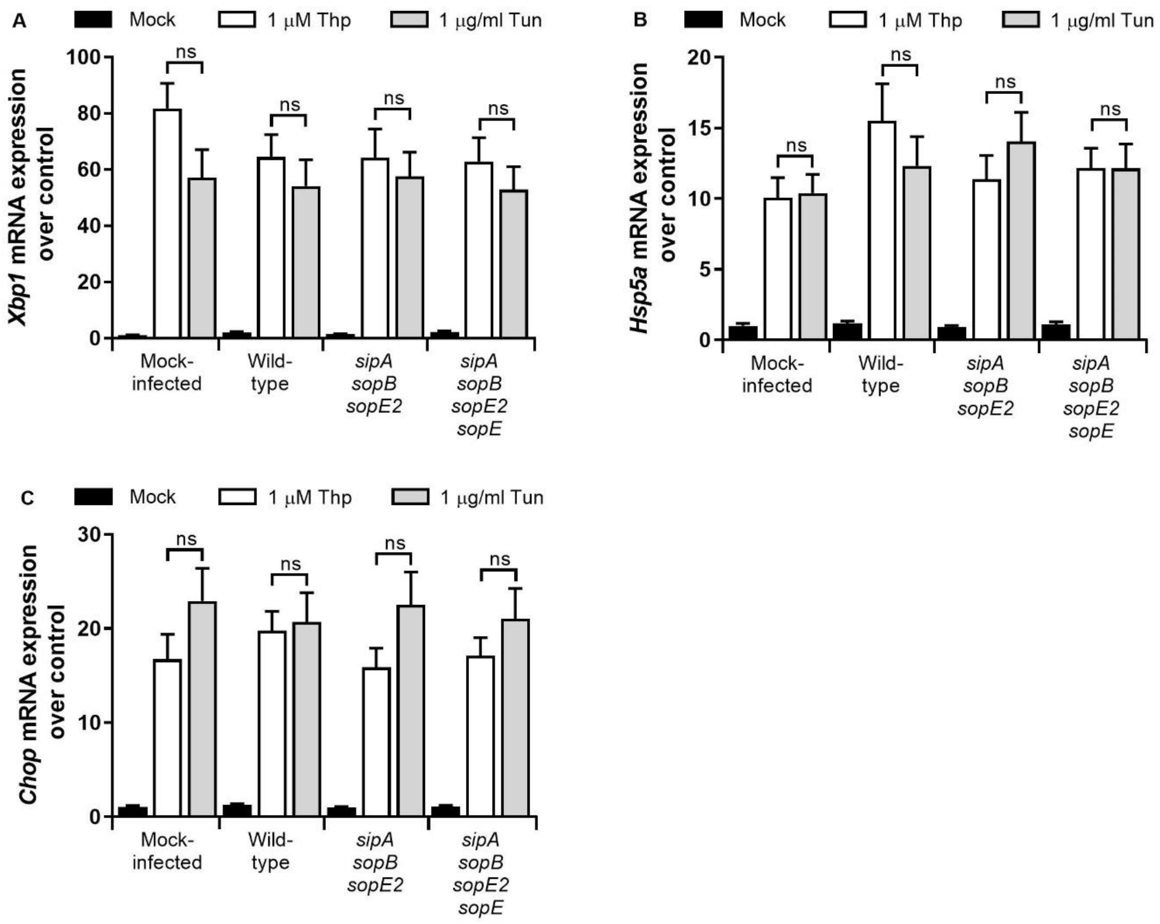

Figure S3. Thapsigargin and tunicamycin induced expression levels of UPR target genes in MODE-K cells. MODE-K cells were pretreated with thapsigargin (1 μM) or tunicamycin (1 μg/ml) and infected with the *S*. Typhimurium wild type strain SL1344, the SopE-positive *sipAsopBsopE2* mutant strain and the *sipAsopBsopE2sopE2* mutant strain. RNA was extracted and RT-PCR performed to determine the expression of (A) *Xbp1*, (B) *Hsp5a* and (C) *Chop*. Data represents means ± standard errors of three independent experiments in duplicate. A two-tailed paired Student *t* test was used to determine statistical significance. A *P* value of <0.05 was taken to be significant.
